# Phenylalanine Ammonia-Lyase: A Core Regulator of Plant Carbon Metabolic Flux Redistribution—From Molecular Mechanisms and Growth Modulation to Stress Adaptability

**DOI:** 10.3390/plants14243811

**Published:** 2025-12-14

**Authors:** Xiaozhu Wu, Suqing Zhu, Lisi He, Gongmin Cheng, Tongjian Li, Wenying Meng, Feng Wen

**Affiliations:** 1 College of Resources & Environment, Jiujiang University, Jiujiang 332000, China; 2 School of Biological Science and Food Engineering, Chuzhou University, Chuzhou 239099, China; 3 College of Pharmacy and Life Sciences, Jiujiang University, Jiujiang 332000, China

**Keywords:** phenylalanine ammonia-lyase, carbon flux reallocation, growth and development, abiotic stresses, biotic challenges

## Abstract

Phenylalanine ammonia-lyase (PAL) is the core branch-point enzyme connecting plant primary aromatic amino acid metabolism to the phenylpropanoid pathway, which determines carbon flux redistribution between growth and defense and is essential for plant adaptation to various environments. Extensive research has clarified PAL’s conserved homotetrameric structure, MIO cofactor-dependent catalytic mechanism, and its roles in plant growth, development, and stress responses. However, there is a lack of comprehensive review studies focusing on PAL-mediated carbon metabolic flux redistribution, specifically covering its structural and evolutionary foundations, the links between this flux regulation and plant growth/development, its multi-layered regulatory network, and its roles in stress adaptation, limiting a comprehensive understanding of its evolutionary and functional diversity. This review systematically covers four core aspects: first, the molecular foundation, encompassing PAL’s structural features and catalytic specificity governed by the MIO cofactor; second, evolutionary diversity spanning from algae to angiosperms, with emphasis on unique regulatory mechanisms and evolutionary significance across lineages; third, the multi-layered regulatory network, integrating transcriptional control, post-translational modifications, epigenetic regulation, and functional crosstalk with phytohormones; and fourth, functional dynamics, which elaborate PAL’s roles in organ development, including root lignification, stem mechanical strength, leaf photoprotection, flower and fruit quality formation, and lifecycle-wide dynamic expression, as well as its mediated stress adaptations and regulatory networks under combined stresses. These insights provide a theoretical basis for targeted manipulation of PAL to optimize crop carbon allocation, thus improving growth performance, enhance stress resilience, and promote sustainable agriculture.

## 1. Introduction

As primary producers, plants rely on dynamic carbon flux partitioning between primary and secondary metabolism to balance growth and defense. Phenylalanine ammonia-lyase (PAL), the rate-limiting enzyme linking aromatic amino acid metabolism to the phenylpropanoid pathway, acts as a key “metabolic valve” directing carbon flux, thereby governing plant adaptation to diverse environments [1]. These photosynthetic assimilates provide not only the essential material basis for plant growth, development, and reproduction but also serve as crucial metabolic reserves that sustain plants under abiotic stresses and biotic challenges. The dynamic partitioning of carbon flux, mediated by PAL, represents a fundamental adaptive strategy that balances the competing demands of growth and environmental resilience. Despite advances in understanding PAL’s structure and functions, critical gaps remain, including its fine-scale carbon flux regulation, integrative roles under combined stresses, and evolutionary diversification across lineages. This review synthesizes current knowledge on PAL-mediated carbon flux reallocation, focusing on its molecular basis, regulatory networks, developmental functions, and stress adaptations, to provide a coherent framework for future research and agricultural applications.

### 1.1. PAL in Plant Carbon Metabolism Networks

**Figure 1:**
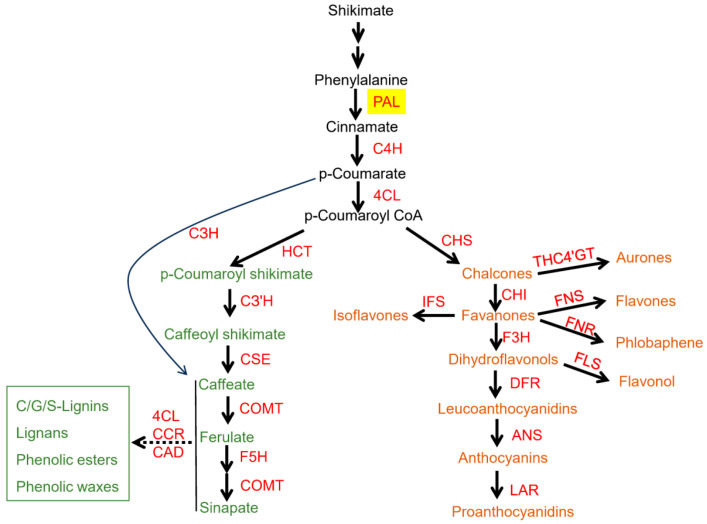
Schematic representation of the phenylpropanoid biosynthetic pathway in plants. PAL, the entry-point and rate-limiting enzyme of this pathway, catalyzes the deamination of L-phenylalanine to trans-cinnamic acid, thereby initiating a cascade of downstream reactions that yield a wide spectrum of phenylpropanoid-derived metabolites. These metabolites include lignins of the guaiacyl (G), syringyl (S), and p-hydroxyphenyl (H) types, as well as lignans, phenolic esters, phenolic waxes, and a variety of flavonoid derivatives such as flavones, flavonols, anthocyanins, proanthocyanidins, isoflavones, and aurones.

The phenylpropanoid pathway, initiated by PAL, is a key secondary metabolic branch that generates lignin, flavonoids, and phytoalexins, which are metabolites essential for structural integrity, photoprotection, and pathogen defense (Figure 1) [2,3,4,5,6]. As the pathway’s entry enzyme, PAL coordinates with downstream enzymes including cinnamate 4-hydroxylase (C4H), 4-coumarate-CoA ligase (4CL) and chalcone synthase (CHS) to maintain metabolic homeostasis [2,7]. For instance, during the seedling stage, carbon is preferentially directed to roots and leaves to support organ establishment, and *PAL* expression (e.g., *AtPAL3/4* in *Arabidopsis thaliana*) remains low to prioritize primary metabolism for cell division in root apical meristems [8,9]. During the vegetative phase, carbon is redirected to stems to enhance structural support, while during the reproductive phase, it is allocated to flowers and fruits, and PAL activity dynamically adjusting to meet organ-specific metabolic demands [10]. Under environmental stress conditions, PAL is rapidly activated, along with other secondary metabolism-related enzymes, and redirects carbon toward the phenylpropanoid pathway to produce defensive compounds that enhance resilience [11,12,13,14,15]. Notably, PAL’s coordination with other branch-point enzymes further refines carbon flux partitioning. For example, phosphoenolpyruvate carboxylase (PEPC) competes with PAL for carbon substrates, with PEPC directing flux toward organic acid synthesis and PAL toward phenylpropanoid production [11,16,17]. Within the phenylpropanoid pathway, PAL exerts crosstalk with competing branch-point enzymes to orchestrate carbon flux diversion. For instance, PAL cooperates with C4H to regulate the phenylpropanoid pathway, which is a typical feedback regulation, where the intermediates cinnamic acid and C4H’s activity, can control the activity of PAL [18,19]. While CHS catalyzes the first committed step of flavonoid biosynthesis by condensing p-coumaroyl-CoA with three molecules of malonyl-CoA to form a chalcone [20,21,22]. These crosstalks ensures that carbon flux is dynamically adjusted to meet plant needs, whether for growth, reproduction, or defense. Regulation of these metabolic “valves” is mediated through multiple, interacting layers of control that collectively position PAL as a central determinant of metabolic flexibility in plants [23,24,25,26,27,28]. At the transcriptional level, regulatory factors such as MYB and WRKY proteins modulate *PAL* gene expression. Notably, the promoters of PAL contain AC elements that confer responsiveness to R2R3-MYB transcription factors [5,29,30]. Post-translational regulation further fine-tunes PAL abundance and activity; in *A. thaliana*, the Kelch repeat F-box proteins KFB01, KFB20, and KFB50 interact with PAL isoforms, promoting their ubiquitination [27]. Epigenetic mechanisms, including DNA methylation and histone modifications, also contribute to PAL regulation. For instance, introduction of a bean *PAL* transgene into tobacco induces unstable epigenetic post-transcriptional silencing [28]. In addition, metabolite-mediated feedback inhibition provides an important metabolic layer of control, allowing plants to adjust PAL activity and carbon flux allocation in response to cellular levels of phenylpropanoid intermediates. Together, these regulatory mechanisms ensure precise modulation of PAL activity and maintain balanced carbon flux partitioning across metabolic networks.

### 1.2. Core Roles of PAL in Carbon Flux Reallocation

Within this carbon metabolism network, PAL occupies a central position due to its unique catalytic role and broad regulatory scope, with its functions spanning molecular, developmental, and stress-related levels. PAL (EC 4.3.1.5) was first purified and identified from barley (*Hordeum vulgare*) seedlings in 1961 [31], and its conserved function as a homotetrameric enzyme with a 4-methylidene-imidazole-5-one (MIO) prosthetic group is conserved across cyanobacteria, algae, bryophytes, and higher plants [7,32,33,34].

First, its molecular and catalytic characteristics lay the foundation for carbon flux regulation. PAL functions exclusively as a homotetramer, with each monomer comprising four functionally distinct domains: an N-terminal extension, a MIO catalytic domain, a core domain, and a C-terminal shielding domain [7]. The MIO prosthetic group, which is formed autocatalytically from an internal Ala-Ser-Gly tripeptide, initiates the deamination of L-phenylalanine via electrophilic attack on the aromatic ring, leading to cleavage of the C–N bond [7,32,33,34]. In *Petroselinum crispum*, the Ser202 residue is essential for MIO formation, and the S202A mutation abolishes enzyme activity [34,35]. In *Anabaena variabilis*, a Q292C mutation promotes intramolecular disulfide bond formation, stabilizing the MIO conformation and markedly increasing catalytic efficiency (*kcat*/*Km*) [36]. Its catalytic specificity, which primarily targets L-phenylalanine in higher plants and exhibiting dual-substrate activity in algae and bryophytes, reflects adaptive evolutionary adjustments. For example, *Photorhabdus luminescens* PAL (PlPAL) catalyzes both L-phenylalanine and L-tyrosine deamination [37], while *Anthoceros agrestis* PAL isoenzymes (AaPAL1 and AaPAL2) have *Km* values of 39 μM and 18 μM for L-phenylalanine, but 3.3 mM and 3.5 mM for L-tyrosine [38]. In *A. thaliana*, AtPAL1, AtPAL2, and AtPAL4 exhibit little to no activity toward L-tyrosine, confirming L-phenylalanine as the physiological substrate for higher plant PALs [9], ensuring carbon flux is effectively channeled into phenylpropanoid metabolism.

Moreover, PAL-mediated carbon flux reallocation supports diverse biological functions. During development, PAL drives root lignification for stress resistance, stem mechanical strength for structural support, leaf flavonoid synthesis for photoprotection, and flower/fruit anthocyanin accumulation for reproductive success. Specifically, under phosphate deficiency, several *OsPAL* family genes in rice are significantly upregulated in roots, promoting lignin accumulation, reducing phosphorus leaching, and improving phosphorus use efficiency [17]. In *Brachypodium distachyon*, RNA interference (RNAi)-mediated suppression of PAL isoforms reduces stem cell wall lignin content by 43% and cell wall-bound ferulic acid by 57%, causing reduced height and lodging [39]. In leaves, low PAL activity in young leaves prioritizes carbon flux for cell expansion and chloroplast formation, while elevated activity in mature leaves promotes flavonoid accumulation for UV photoprotection [8,40,41]. In colored cultivars of apple and myrtle berry, PAL activity correlates positively with flavonoid and anthocyanin accumulation, critical for pollinator attraction and seed dispersal [42,43,44,45]. Under stress, PAL redirects carbon to lignin (physical barriers) and flavonoids/phytoalexins (chemical defense), enhancing resilience to abiotic and biotic challenges. Overexpression of *PePAL* in moso bamboo (*Phyllostachys edulis*) substantially increases stem lignin content, enhancing compressive strength [3]; *GhPAL9* in cotton (*Gossypium hirsutum*) is upregulated upon infection with *Verticillium dahliae*, inducing root and stem lignification and reducing pathogen colonization [46]. Overexpression of *GuPAL1* from licorice (*Glycyrrhiza uralensis*) in *A. thaliana* increases flavonoid content, enhancing drought tolerance [10]; in *Ammopiptanthus mongolicus*, cold-induced *PAL* gene expression promotes flavonoid accumulation and reduces leaf freezing injury [47]. However, PAL does not act in isolation, its coordination with downstream enzymes (e.g., C4H, CHS) determines flux priority, while its integration with key hormone signaling pathways (ABA, SA, and methyl jasmonate (MeJA)) modulates stress-responsive metabolic adjustments. Despite these insights, gaps remain in understanding how PAL fine-tunes carbon flux quantitatively, integrates signals under combined stresses, and evolves across plant lineages, and these gaps are what this review aims to address.

Collectively, PAL serves as a central metabolic hub between primary metabolism and phenylpropanoid secondary metabolism, integrating structural, catalytic, and regulatory mechanisms to balance plant growth, development, and stress adaptation. Recent advances in molecular biology, structural biology, and metabolomics have greatly deepened our understanding of PAL’s structure (conserved homotetrameric architecture), catalytic mechanism (MIO prosthetic group-dependent), functional diversity (in organ development like root lignification and flower/fruit quality formation), and evolutionary dynamics, as well as its pivotal roles in stress responses [32,33,34,46]. Nevertheless, key gaps remain regarding fine-scale carbon flux regulation (e.g., quantitative coordination with downstream enzymes), integrative functions under combined stresses, and cross-lineage evolutionary drivers [32], leading to fragmented knowledge that limits crop improvement strategies. As a central hub coordinating plant growth, development, and stress adaptation, this review addresses these gaps by systematically organizing research around four interconnected themes—molecular/evolutionary foundations, multi-layered regulatory networks, developmental roles via carbon flux partitioning, and stress adaptive functions (including combined stresses)—to provide a coherent framework for future studies on carbon flux regulation and crop metabolic engineering.

## 2. Structural and Evolutionary Foundations of PAL-Mediated Carbon Flux Regulation

The ability of PAL to direct metabolic flow from primary metabolism toward the phenylpropanoid pathway is governed by three closely interconnected molecular characteristics: a highly conserved homotetrameric protein structure that ensures catalytic stability and efficiency, a substrate-specific catalytic mechanism centered on the MIO prosthetic group, and evolutionary diversification of the PAL gene family through copy number expansion and lineage-specific structural variation, which enables spatiotemporal regulation of expression. Collectively, these molecular features form the molecular foundation for dynamic carbon flux allocation, allowing plants to balance growth, defense, and environmental adaptation with remarkable flexibility.

### 2.1. Structural Basis of PAL Catalysis and Species-Specific Functional Adaptation

**Figure 2:**
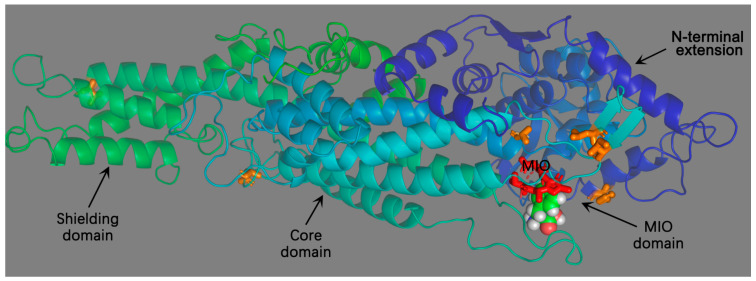
Structure of PAL Monomer from Parsley. The ribbon representation of PcPAL is re-built based on the coordinates of PAL (1W27) via the PyMol program [7].

PAL displays an evolutionarily conserved protein structure across a broad phylogenetic range, from prokaryotes to higher plants, and functions exclusively as a homotetramer. Each monomer comprises four functionally distinct domains: an N-terminal extension, a MIO catalytic domain, a core domain, and a C-terminal shielding domain (Figure 2). The coordinated interaction between key active-site residues, such as Ser, His, and Lys, and the MIO prosthetic group directly determines PAL’s catalytic efficiency and substrate specificity, providing the structural foundation for efficient carbon channeling into the phenylpropanoid pathway [7,32].

Formation of the homotetramer is indispensable for PAL enzymatic activity. The composition of each subunit, the spatial organization of catalytic residues, and inter-subunit interactions jointly modulate substrate binding, MIO prosthetic group formation, and product release. The four domains of PAL monomers perform distinct, non-redundant roles: (1) N-terminal extension (24–150 amino acids). This region is typically a flexible polypeptide segment that stabilizes interfaces between adjacent domains and facilitates homotetramer assembly. Deletion of this region disrupts oligomerization and drastically reduces catalytic activity. For example, in *P. crispum* PAL, removal of residues 1–24 destabilizes the tetramer and decreases enzymatic activity to less than 2% of the wild-type level [7]. (2) MIO catalytic domain (200–260 amino acids): This domain centers on a conserved Ala-Ser-Gly tripeptide, which undergoes autocatalytic dehydration to form the MIO prosthetic group. The Ser residue is essential for MIO generation; mutation of Ser202 to Ala (S202A) in parsley PAL completely abolishes cofactor formation and eliminates enzyme activity [7,34,35]. (3) Core domain (250–300 amino acids): This domain forms a hydrophobic substrate-binding pocket, where residues such as Lys and His interact with the aromatic ring of L-phenylalanine through cation–π interactions and hydrogen bonding, thereby determining substrate specificity [32]. (4) C-terminal shielding domain (~120 amino acids, present only in higher plants): This domain is primarily composed of α-helices, forming an arch-like structure that covers the catalytic center, restricts the entry of non-specific substrates, and protects the active residues from nucleophilic attack [7].

Species-specific variations in tetramer assembly and domain composition underpin the functional diversification of PAL. For instance, in the cyanobacterium *Anabaena flos-aquae*, a Q292C mutation in the MIO catalytic domain enhances disulfide bond formation between subunits, stabilizing the homotetramer and improving catalytic performance under high-salinity conditions [36,48]. In *A. thaliana*, AtPAL1/2/4 contain unusually long N-terminal extensions (~145 amino acids) enriched in hydrophobic residues, which strengthen inter-subunit hydrophobic interactions and enhance structural stability [9]. Similarly, PAL enzymes isolated from agricultural residues such as wheat straw and peanut shells exhibit a high surface abundance of proline residues that reinforce hydrophobic packing and confer superior thermal stability, by contrast, microbial PALs such as those from *Rhodotorula glutinis* show significantly lower thermostability [49].

PAL catalyzes the non-oxidative deamination of L-phenylalanine to yield trans-cinnamic acid and ammonia, representing the gateway reaction that governs the entry of carbon into the phenylpropanoid pathway. This catalytic process exhibits notable interspecies variability [7,49]. Most plant PALs demonstrate high specificity for L-phenylalanine, with minimal cross-reactivity toward L-tyrosine, a property determined by the geometric constraints of the substrate-binding pocket and the electronic characteristics of the aromatic ring. For example, *Arabidopsis* PAL isoforms exhibit *Km* values of 64–71 μM for L-phenylalanine, whereas their *Km* values for L-tyrosine are substantially higher, resulting in significantly reduced catalytic efficiency [9]. In contrast, some algae and microbial PALs display dual-substrate activity. For instance, AaPAL2 from *A. agrestis* has a *Km* of 18 μM for L-phenylalanine and 3.5 mM for L-tyrosine, reflecting relaxed substrate discrimination [38].

Kinetic parameters of PAL, including *Km*, *kcat*, and optimal pH/temperature, exhibit considerable interspecies variability, reflecting adaptive evolution to distinct ecological niches. Higher plant PALs typically possess broader pH and temperature tolerance ranges and higher catalytic efficiency than those from lower plants. Reported *Km* values for L-phenylalanine range from 18 μM to 1.07 mM, and *kcat* values from 0.109 s^−1^ to 1117 min^−1^. In general, higher plant PALs have lower *Km* values, indicating stronger substrate affinity compared with PALs from primitive lineages [38,49,50,51].

### 2.2. Evolutionary Expansion and Structural Diversification of the PAL Gene Family

The PAL gene family typically exists as a multi-copy family in plant genomes and exhibits substantial variation in copy number and exon–intron structure among different plant lineages. This genetic diversity provides the molecular basis for adaptive responses to various environmental challenges.

**Figure 3:**
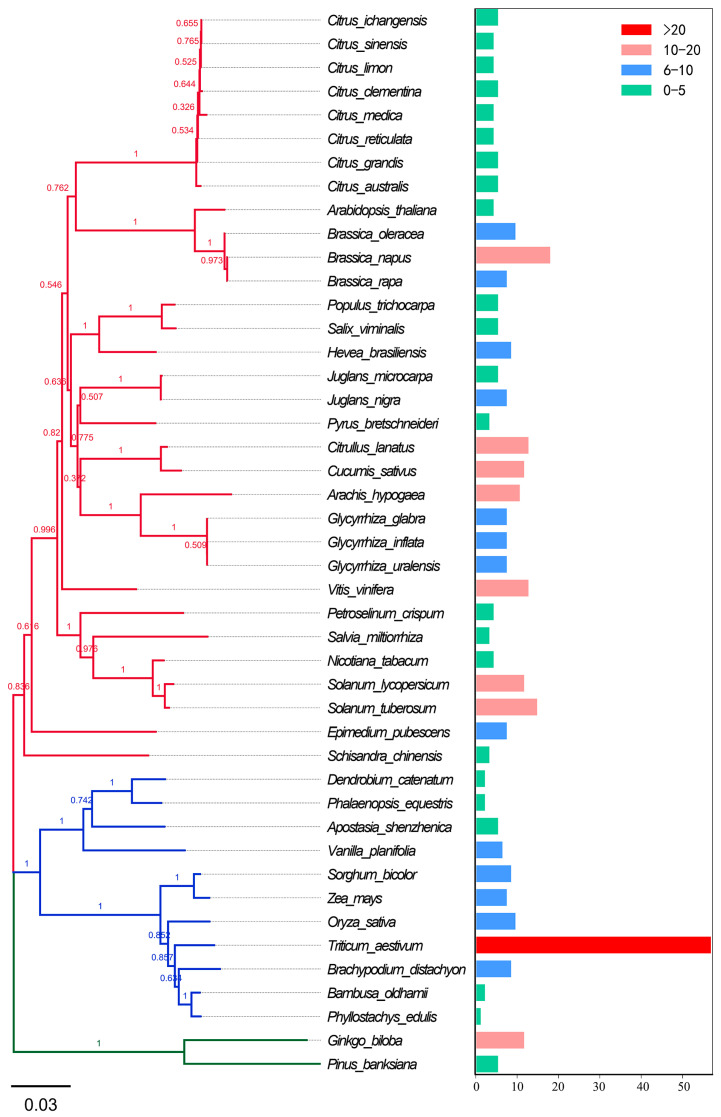
Phylogenetic distribution and gene copy number variation of the PAL family across representative plant species. The phylogenetic tree illustrates the evolutionary relationships among plant lineages harboring *PAL* genes, with branch color coding for plant taxonomic groups: red branches represent dicotyledons, blue branches represent monocotyledons, and green branches represent gymnosperms. The accompanying bar chart depicts the corresponding *PAL* gene copy numbers, with color coding indicates gene abundance: red (>20 genes), light red (10–20 genes), blue (6–10 genes), and green (0–5 genes).

**Figure 4:**
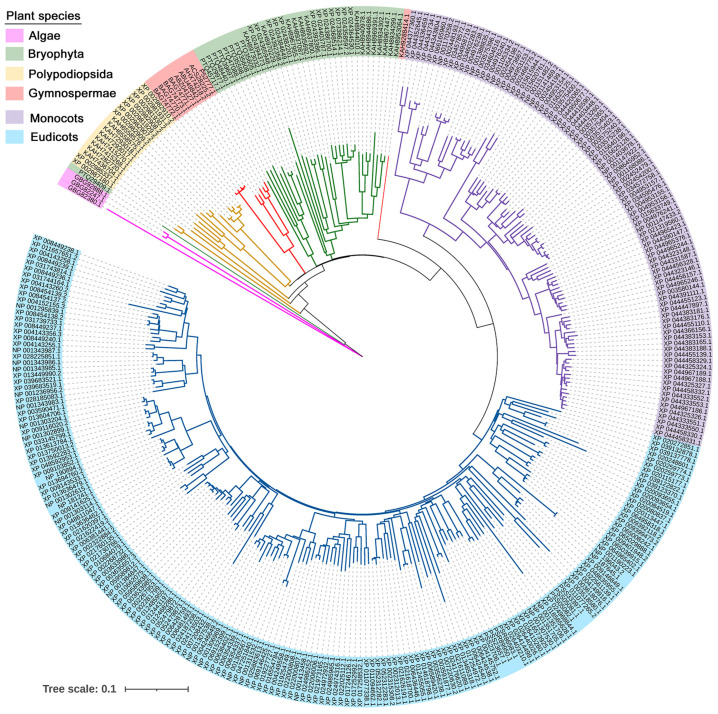
Phylogenetic tree of PAL homologs retrieved from Genbank database. The unroot tree contains 333 proteins from Algae to Angiosperm totally 58 plant species and different colors indicate different plant stages.

Expansion of PAL gene family copy number is closely aligned with plant evolutionary advancement and is accompanied by progressive functional specialization (Figure 3 and Figure 4). As summarized in Supplementary Table S1, the copy number of PAL genes varies significantly across different plant lineages, reflecting their evolutionary history. Algae usually contain only one to two *PAL* gene copies, which display conserved and relatively simple functions mainly associated with basal phenylpropanoid metabolism, such as the synthesis of simple phenolic acids for primary defense. Bryophytes exhibit unique PAL functional characteristics distinct from algae, their PAL not only participates in synthesizing simple phenolic acids for primary defense but also plays a pivotal role in adapting to the transition from aquatic to terrestrial environments, such as UVB and pathogen attack [52]. Gymnosperms (e.g., pine, ginkgo) generally possess three to five PAL copies that already show preliminary functional partitioning between lignin biosynthesis and stress response [53]. In angiosperms, the copy number of *PAL* genes increases markedly, commonly ranging from three to fifteen copies. This expansion is primarily driven by genomic events including whole-genome duplication (WGD), tandem duplication, and transposon-mediated segmental duplication [54,55]. Moreover, different *PAL* paralogs in angiosperms have undergone extensive functional specialization, forming a coordinated regulatory network that mediates the growth–defense trade-off. This specialization enables tissue- and developmental stage-specific expression of *PAL* genes, thereby facilitating carbon flux allocation between growth-related and defense-related pathways. For example, *Arabidopsis AtPAL1/2* double mutants exhibit hypersensitivity to environmental stress, with lignin content in flowers and stems reduced to approximately 30% of that in wild-type plants, confirming their essential role in growth-associated lignin biosynthesis [9,56]. Conversely, overexpression of *PAL* genes, such as *NtPAL* in tobacco and *PvPAL3* in common bean, generally enhances carbon flux into phenylpropanoid pathway, which, in turn, leads to increased accumulation of phenylpropanoid-derived defense metabolites and improved resistance to microbial pathogens [57,58].

The genomic structure of *PAL* genes remains evolutionarily conserved across most plant taxa. The majority harbor two exons and a single intron, with intron lengths typically ranging from 500 to 2000 bp. This structural conservation is thought to enable rapid transcription initiation and efficient pre-mRNA splicing, ensuring timely adjustments in PAL activity and carbon flux distribution under fluctuating physiological conditions. Nonetheless, lineage-specific structural variations have been identified, reflecting adaptive evolution to distinct ecological niches [54,59,60]. For example, all *PAL* genes in peanut (*Arachis hypogaea*) maintain the canonical two-exon–one-intron structure without intron gain or loss, suggesting strong selective pressure to preserve this genomic organization [61]. In *Epimedium* species, *EpPAL2* and *EpPAL3* share low sequence identity with other *EpPAL* members and contain two unique introns, a rare feature among *PAL* genes. This structural divergence suggests their origin from an independent ancestral lineage and potential specialization in drought or herbivore defense [54]. Rare cases of intron loss have also been reported, such as *BoPAL1* from *Bambusa oldhamii*, the first identified intronless *PAL* gene in angiosperms. Its 5′ flanking region contains light-responsive cis-elements, such as P-box and GT-1 motif, implying that intron loss may eliminate the need for pre-mRNA splicing, accelerating transcriptional activation in response to light signals and enabling rapid redirection of carbon flux to flavonoid synthesis for photoprotection [60].

In summary, the evolutionary conservation of PAL’s protein and gene structure ensures the stable inheritance of its core catalytic function, whereas lineage-specific structural innovations and copy number diversification confer molecular flexibility for adaptive specialization. The interplay between conservation and diversification establishes a finely tuned regulatory framework that allows PAL to modulate carbon flux within complex metabolic networks, thereby coordinating plant growth, development, and stress resilience.

## 3. Regulation of Plant Growth and Development by PAL-Mediated Carbon Flux Reallocation

The directional redistribution of carbon flux from primary metabolism to the phenylpropanoid pathway, mediated by PAL, not only provides structural polymers and signaling molecules essential for plant growth and development but also modulates growth decisions through the regulation of the carbon–nitrogen balance. This section outlines the mechanisms by which PAL-mediated carbon flux reallocation regulates plant growth and developmental processes from two complementary perspectives, the regulation of organ development and the coordination of carbon–nitrogen metabolism. It also underscores PAL’s central role in optimizing “source–sink” carbon allocation and shaping adaptive growth strategies in plants.

### 3.1. Association Between Carbon Flux Reallocation and Plant Organ Development & Lifecycle-Wide Dynamic Regulation

The development of both vegetative (roots, stems, leaves) and reproductive (flowers, fruits) organs, as well as the adaptive responses throughout the plant lifecycle, are dependent on the precise partitioning of PAL-mediated carbon flux into distinct branches of the phenylpropanoid pathway. Lignin confers key mechanical support and cell wall rigidity, flavonoids function as modulators of hormone signaling and redox homeostasis, and anthocyanins facilitate reproductive success by visually attracting pollinators. These metabolites constitute a coordinated functional network that integrates “structural reinforcement, signaling modulation, and adaptive defense” [3,10,47,54,62,63,64]. Meanwhile, the dynamic expression and functional plasticity of PAL across developmental stages ensure the spatiotemporal allocation of carbon flux to fulfill stage-specific metabolic demands, with a priority on growth establishment during early phases and defense or reproduction in later stages.

Roots. Root elongation, lateral root initiation, and stress tolerance are strongly influenced by PAL-dependent allocation of carbon toward lignin and flavonoid biosynthesis. Lignin deposition strengthens the mechanical structure and barrier properties of root cell walls, whereas flavonoids modulate auxin gradients by inhibiting root-specific auxin efflux transporters, directly affecting root architecture and branching [14,39,65,66,67]. For example, NaCl stress significantly induces the transcription of *AmPAL* in *Astragalus membranaceus*, with the highest expression detected in roots. Overexpression of *AmPAL* in tobacco (*Nicotiana tabacum*), resulted in enhanced PAL enzyme activity under salt stress, reduced malondialdehyde (MDA) levels, and significantly greater root elongation compared to wild-type plants, confirming that PAL-mediated carbon reallocation contributes to improved salt-stress adaptation in roots [14].

Stems. Stem mechanical strength, vascular transport efficiency, and lodging resistance are primarily determined by PAL-directed carbon flux into lignin biosynthesis, as lignin content and monomeric composition dictate stem rigidity [3]. In *P. edulis*, the transcript levels of most *PePAL* genes (except *PePAL11*) progressively increase during stem development, peak during maximum lignification, and subsequently stabilize or decline, indicating that *PePAL* genes are key regulators of secondary wall formation [3]. Similarly, overexpression of *PAL* genes in wheat (*Triticum aestivum*), castor bean (*Ricinus communis*), and fritillary (*Fritillaria thunbergii*) significantly enhances stem lignin content, thereby improving mechanical strength and reducing lodging risk [51,68,69].

Leaves. Leaf expansion, photoprotection, and senescence are also closely regulated by PAL-mediated carbon flux allocation. In young leaves of *Vaccinium dunalianum* and *Populus* species, *PAL* expression is significantly higher than in mature leaves, accompanied by increased chlorogenic acid accumulation, suggesting that active phenylpropanoid metabolism supports early leaf development [70,71,72]. In mature leaves, PAL-mediated carbon flux is preferentially directed to flavonoid biosynthesis to enhance UV photoprotection and antioxidant defense, with a smaller fraction allocated to lignin production for maintaining leaf structure. During senescence, elevated PAL activity promotes flavonoid accumulation that scavenges ROS and delays aging. In the anti-senescence rice variety YN, genes encoding *PAL*, *C4H*, *4CL*, and *CHS* are upregulated, leading to higher flavonoid accumulation and delayed leaf senescence [73]. In raspberry (*Rubus idaeus*), prematurely senescent leaves exhibit reduced chlorogenic acid content but elevated naringenin chalcone levels, indicating that PAL-mediated carbon partitioning between these metabolites influences leaf aging dynamics [74].

Flowers and Fruits. The development of reproductive organs, pollen fertility, and fruit quality are tightly governed by PAL-mediated carbon flux reallocation. Anthocyanins determine floral pigmentation and pollinator attraction [75,76,77], while flavonoids regulate pollen germination and tube growth, both processes critically dependent on PAL activity [78,79,80]. For example, in broccoli (*Brassica oleracea*) anthers, PAL activity correlated positively with pollen fertility, underscoring its importance in reproductive development [81]. In fruits, PAL plays a pivotal role in pigmentation, antioxidant capacity, and abscission regulation. In melon (*Cucumis melo*), *PAL* expression increases during ripening, promoting phenylpropanoid metabolism and enhancing antioxidant potential [82]. Similarly, in wolfberry (*Lycium barbarum*), the activities of PAL, CHS, and F3H increase during fruit maturation, leading to flavonoid accumulation [83]. In *Fragaria chiloensis*, abscisic acid (ABA) treatment induces *FcPAL*, *FcCHS*, and *FcANS* expression, accelerating anthocyanin biosynthesis and fruit coloration [84]. PAL also contributes to fruit abscission control: in citrus, *PAL* expression in the abscission zone of mature fruits rises sharply within 72 h of abscission onset, while immature fruits show minimal PAL transcription, indicating a key role for PAL-mediated carbon redistribution in fruit detachment processes [85].

Lifecycle-Wide Dynamic Expression. PAL exhibits dynamic changes in expression patterns and functional roles throughout the plant lifecycle, which are tightly tailored to the metabolic requirements of distinct developmental stages [71,86]. At the seedling stage, PAL function is prioritized toward root lignification and stress adaptation. In wheat (*Triticum aestivum*), *TaPAL33* is significantly upregulated under high-temperature stress, increasing lignin content to enhance stress tolerance [87]. Similarly, in rice, the transcription of multiple *OsPAL* genes in shoots and roots is upregulated under phosphorus deficiency, and the resultant phenolic compounds solubilize soil phosphorus to promote seedling establishment [17]. During the vegetative growth stage, PAL function shifts to enhancing stem mechanical support. In moso bamboo, *PePAL* transcription levels and enzyme activity are upregulated during the early growth stage of bamboo shoots, likely reflecting their activation to supply substantial amounts of cinnamic acid, the initial metabolite of the phenylpropanoid pathway, for downstream lignin biosynthesis [88,89]. At the reproductive growth stage, PAL mediates flower pigmentation and fruit quality development by regulating downstream metabolite synthesis. Anthocyanins, as key downstream metabolites of the PAL-initiated phenylpropanoid pathway, are the most prevalent plant pigments responsible for flower coloration to attract pollinators [90,91]. In tomato, SlPAL4 and SlPAL6 are functionally prominent during fruit ripening, contributing to phenolic accumulation and flavor improvement [92]. Notably, some PAL homologs exhibit pleiotropic roles across multiple lifecycle stages rather than stage-specific functions. For instance, *PAL* genes in *Vaccinium dunalianum* are implicated in both leaf vegetative growth and sexual reproduction, including flower bud development [71]. This dynamic shift in *PAL* expression and functional specialization ensures that carbon flux is preferentially allocated to growth-related processes in early developmental stages and defense- or reproduction-related metabolism in later stages, thereby maintaining metabolic homeostasis throughout the plant lifecycle.

### 3.2. Synergistic Regulation of PAL and Other Pathways: Carbon–Nitrogen Balance

Plant growth and development depend on the dynamic coordination between carbon and nitrogen metabolism. As a key enzyme linking primary carbon metabolism with secondary phenylpropanoid metabolism, PAL integrates signals from carbon availability and nitrogen status, thereby serving as a metabolic node that balances resource allocation.

PAL belongs to the class of carbon–nitrogen lyases that catalyze double-bond formation. During catalysis, PAL converts L-phenylalanine into trans-cinnamic acid and an amino-enzyme intermediate, which subsequently releases ammonia. The liberated ammonia is rapidly reassimilated into the nitrogen pool through the action of glutamine synthetase [93]. From a metabolic perspective, the synthesis of L-phenylalanine itself requires nitrogen derived from primary metabolites such as glutamate and aspartate; thus, nitrogen availability directly affects substrate supply for PAL and consequently regulates carbon flux toward the phenylpropanoid pathway. Conversely, enhanced PAL activity consumes L-phenylalanine and modifies nitrogen partitioning between primary and secondary metabolism [94,95,96].

Under low-nitrogen conditions, plants often upregulate *PAL* expression to activate phenylpropanoid metabolism. This strategy serves two main purposes: PAL facilitates nitrogen recycling by the release of ammonia during L-phenylalanine deamination and redirects carbon flux toward secondary metabolite synthesis [95,96]. For example, in *Cornus officinalis*, nitrogen deficiency induces PAL activity and upregulates *PAL*, *CHS*, and isoflavone synthase genes, leading to increased accumulation of phenolic compounds [96]. Similarly, in *Labisia pumila*, nitrogen limitation enhances PAL activity, correlating with elevated total flavonoid (TF) and total phenolic (TP) levels. This PAL activation reduces soluble protein synthesis by 15–20% and increases free L-phenylalanine content, shifting metabolic investment toward carbon-based secondary metabolites (CBSMs) [95].

In contrast, high nitrogen availability favors amino acid and protein biosynthesis, channeling nitrogen into primary metabolism while suppressing PAL activity and secondary metabolite production [97,98,99,100]. For instance, nitrogen fertilization reduces PAL activity in rice, leading to decreased resistance to *Magnaporthe oryzae* [101]. Similar effects have been observed in *Medicago truncatula*, where nitrogen fertilization decreases resistance to *Aphanomyces euteiches*; and in soybean and subarctic tundra vegetation, where nitrogen enrichment lowers the accumulation of defensive phenolics such as coumarins, total phenolics, and condensed tannins [102,103,104]. In roots of *Fagus sylvatica* and *Picea abies*, increased nitrogen availability decreases antifungal phenolics such as epicatechin and resveratrol, with only minor increases in protocatechuic acid [105]. Collectively, these findings indicate that nitrogen fertilization typically suppresses PAL activity, reduces the biosynthesis of defensive secondary metabolites, and compromises disease resistance. This pattern is consistent across diverse crop–pathogen systems, including rice susceptibility to *Sarocladium oryzae* (sheath rot), grapevine (*Vitis vinifera*) to *Uncinula necator* (powdery mildew), potato (*Solanum tuberosum*) to *Phytophthora infestans* (late blight), and apple (*Malus domestica*) to *Venturia inaequalis* (apple scab) [106,107,108,109].

## 4. PAL-Mediated Carbon Flux Reallocation and Plant Stress Adaptation

PAL represents a central enzymatic node in plant stress defense systems. By dynamically reallocating carbon flux, PAL channels photosynthetically fixed carbon toward the biosynthesis of flavonoids, lignin, and phytoalexins, thereby constructing both physical barriers and chemical defense frameworks. In parallel, through extensive metabolic crosstalk with other pathways, PAL contributes to maintaining cellular redox equilibrium and enhances plant resilience to a wide range of abiotic and biotic stresses, including drought, salinity, pathogen attack, and heavy-metal toxicity [110,111,112,113]. This section examines the mechanisms by which PAL-mediated carbon flux redistribution supports plant adaptation to abiotic and biotic stresses, emphasizing its function as a regulatory hub in “carbon flux redirection–stress adaptation response”.

### 4.1. PAL-Mediated Carbon Flux Regulation and Responses to Abiotic Stress

Abiotic stresses such as drought, salinity, extreme temperature, and heavy-metal exposure disrupt membrane integrity, inhibit metabolic enzymes, and promote overproduction of ROS, collectively threatening plant survival. PAL mitigates these adverse effects by channeling carbon into the phenylpropanoid pathway to generate three categories of protective metabolites: flavonoids (which scavenge ROS), lignin (which reinforces cell walls), and phenolic acids (which chelate heavy metals). Together, these metabolites constitute a multidimensional defense network that alleviates abiotic stress damage [114,115,116,117,118].

Drought and salinity stress. Both drought and salt stress induce osmotic imbalance and ionic toxicity. PAL mitigates these effects through osmotic regulation and structural fortification. Flavonoids act as osmoprotectants that preserve cellular turgor and neutralize ROS, while lignin deposition enhances root barrier function and reduces uncontrolled ion influx [119,120,121,122]. Under drought stress, the phytohormone ABA signaling activates the *PAL* expression, redirecting carbon flux toward flavonoid biosynthesis. These flavonoids maintain cellular water balance by modulating osmotic pressure and safeguard the photosynthetic machinery from oxidative damage, while lignin accumulation reinforces vascular tissues for more efficient water transport. For example, in *Brassica napus*, drought stress markedly increased PAL activity, flavonoid content, leaf relative water content, and stem lignification, collectively improving drought tolerance [117,118,123,124,125]. Comparable responses were observed in sea buckthorn (*Hippophae rhamnoides*) and tea (*Camellia sinensis*), where drought stress enhanced PAL-mediated accumulation of naringenin and dihydroquercetin together with elevated expression of *F3H* and *FLS* [126,127]. In *Ginkgo biloba* leaves, drought stress upregulated *PAL1* and *PAL2* expression, resulting in higher concentrations of catechin, *p*-coumaric acid, and naringenin [128]. Under salinity stress, PAL activity promotes flavonoid-mediated membrane stabilization and induces lignin deposition in the root endodermis to limit Na^+^ uptake. For example, elevated PAL activity in *B. napus* and PAL-driven lignification of Casparian strips in *Cucumis sativus* both restricted Na^+^ entry into the stele and improved salt tolerance [129,130,131].

Temperature stress. Extreme temperatures impose contrasting physiological pressures. For instance, chilling stress disrupts membrane fluidity and organelle function, whereas heat stress damages photosystems and accelerates enzyme inactivation [132,133,134]. PAL responds to these conditions through differential carbon-flux partitioning. Under low temperature, carbon is preferentially directed toward lignin biosynthesis to strengthen cell walls and membranes, while heat stress favors flavonoid synthesis that shields photosystems from ROS injury [132,135,136,137]. Chilling-induced PAL activation increases lignin deposition and enhances ROS detoxification by flavonoids [138,139]. For instance, cold acclimation in cucumber fruits up-regulated the expression of *PAL*, *4CL*, and *CCR*, increasing lignin and flavonoid accumulation and improving cold tolerance during storage [137]. In poplar stems and wheat roots, low temperature triggered lignin accumulation, whereas wheat leaves displayed reduced lignin content, indicating organ-specific modulation of PAL-mediated carbon flux [140,141]. In winter wheat, chilling stress reduced leaf PAL activity but increased the accumulation of free L-phenylalanine and soluble phenolics, implying that substrate accumulation rather than enzyme activation, contributes to cold tolerance [141]. In bamboo shoots, high storage temperatures accelerated lignin and cellulose deposition; PAL and CAD activities increased continuously during storage, correlating positively with lignin content [142]. Under heat stress, PAL-derived flavonoids remove ROS and alleviate photoinhibition, yet prolonged heat may lower PAL catalytic efficiency (reduced *Vₘₐₓ*) and hasten flavonoid degradation [143,144,145]. For example, in *Scutellaria baicalensis*, sustained heat reduced PAL activity and baicalin/baicalein accumulation [146], whereas moderate heat acclimation transiently induced *PAL* expression, as observed in citrus fruits [147,148,149,150] and grapevine [151], where salicylic acid (SA) signaling elevated PAL activity and improved thermotolerance.

Heavy metal stress. Heavy-metal exposure interferes with enzymatic function and intensifies oxidative stress. PAL mitigates such toxicity by reallocating carbon toward phenolic acids and flavonoids that perform dual chelation and antioxidation roles. These compounds bind heavy-metal ions, limiting their cellular interactions, and concurrently neutralize ROS to preserve redox balance [152,153,154]. For example, in soybean, Pb^2+^ treatment enhanced PAL transcription and activity; in buckwheat (*Fagopyrum esculentum*), Al stress similarly increased PAL activity and phenolic accumulation [155,156]. In wheat and buckwheat seeds, SA pretreatment counteracted Cd-induced inhibition of PAL, boosted lignin and flavonoid synthesis, and reduced Cd toxicity [157]. In *Aegilops tauschii*, Cd^2+^ stress activated *AetPAL* expression; the regulatory protein AetSRG1 stabilized PAL through phosphorylation and promoted SA biosynthesis, jointly enhancing Cd tolerance [26].

### 4.2. PAL-Mediated Carbon Flux and Defense Responses to Biotic Stress

Pathogenic microbes and herbivorous insects challenge plant fitness by invading tissues, depleting nutrients, and secreting toxins. PAL counters these biotic threats by channeling carbon flux into the production of phytoalexins, lignin, and volatile phenylpropanoids, establishing multilayered defense systems. Simultaneously, PAL integrates with hormonal signaling pathways to trigger downstream defense gene expression, reinforcing both structural and chemical barriers [27,63,158,159,160].

Pathogen stress. Infection by fungi or bacteria consistently induces PAL-dependent metabolic redirection. PAL-driven phytoalexins suppress pathogen growth, while lignin deposition at infection sites forms structural barriers that confine invasion [161,162]. This defense mechanism is evolutionarily conserved and transcriptionally regulated. For instance, the *Verticillium dahliae* effector PevD1 activates *Arabidopsis* ERF114, which directly binds the *PAL1* promoter to induce its expression; during *Pseudomonas syringae* pv. tomato (Pst DC3000) infection, ERF114 similarly enhances *PAL1* transcription and resistance, confirming ERF-mediated PAL activation as a broad regulatory module [163] PAL also contributes to SA biosynthesis. In rice, SA accumulation depends mainly on the PAL pathway, with OsMYB30 directly binding the promoters of *OsPAL6* and *OsPAL8* to drive SA accumulation and disease resistance. In *A. thaliana*, although the isochorismate synthase (ICS) pathway contributes 90% of SA production, PAL-derived phenolic acid precursors still modulate SA homeostasis, providing an important complementary route for SA biosynthesis [164,165].

Patterns of carbon-flux allocation differ with pathogen type. Under fungal attack, PAL redirects flux toward lignin and phenolic acid synthesis to counter fungal cell-wall-degrading enzymes. For instance, selenium nanoparticle priming in maize (*Zea mays*) against *Fusarium* enhanced PAL activity, diverted flux from flavonoids to phenolic acids (caffeic, ferulic acids) and lignin precursors, thickened cell walls, and accumulated antifungal metabolites to enhance the defense ability [163,166]. In contrast, bacterial infection requires faster defense responses, so PAL-mediated carbon flux is preferentially directed toward phytoalexins with broad-spectrum antibacterial activity. In *A. thaliana*, bacterial challenge upregulated *PAL1* and *PAL2*, while *pal* mutants exhibited reduced lignin and SA content and increased susceptibility [56,167]. PAL-derived phenylpropanoids are essential precursors of diverse phytoalexins [161,168]. In tobacco, infection by *Ralstonia solanacearum* upregulated PAL activity and flavonoid accumulation, confirming defense-oriented carbon flux reallocation [169,170].

Herbivory stress. Feeding by herbivorous insects, such as aphids and lepidopteran larvae, activates PAL-mediated defenses that channel L-phenylalanine toward cinnamic acid, subsequently converted into phenolic acids and flavonoids. These metabolites function in both direct and indirect defenses—reducing palatability, inhibiting digestive enzymes, and promoting volatile signals that attract natural enemies of herbivores [171,172,173,174]. In direct defense, phenolic quinones formed by oxidation of phenolics in tomato leaves covalently bind to proteins, impairing protein digestion in *Helicoverpa armigera* and *Spodoptera exigua* and exhibiting direct toxicity [175,176]. Flavonoids also damage insect midgut epithelial cells: catechin extracted from *Artocarpus lacucha* exhibited strong insecticidal activity against *Spodoptera litura*, with a 24 h LD_50_ of ~8.37 μg per larva, and inhibited the activity of acetylcholinesterase, carboxylesterase, and glutathione S-transferase [177]. Similarly, apigenin-rich extracts of *Acorus calamus* were toxic to *S. litura* [178]. In indirect defense, plants release volatile phenylpropanoids that act as signals to attract parasitoids and predators of herbivores. These volatile organic compounds (VOCs), together with terpenes and green leaf volatiles, form complex chemical cues that guide beneficial insects to locate their herbivorous prey. Methyl salicylate, for example, mediates species-specific effects on insect–host interactions, by deterring *Rhopalosiphum padi* and *Aphis fabae* from host selection [179,180].

### 4.3. Regulatory Network of PAL and Phytohormones Under Combined Stresses

Combined stresses are widespread in natural environments and are generally more detrimental to plant performance than individual stress factors. The PAL-initiated phenylpropanoid pathway forms complex regulatory networks with multiple phytohormones, with ABA acting as a central integrator of environmental cues to optimize carbon-flux reallocation and enhance plant tolerance to multi-stress conditions. ABA is a major regulator of plant responses to drought, salinity, and cold, and its interaction with PAL is essential for redirecting carbon flow toward metabolites that mitigate stress-induced damage. Under combined stress, ABA accumulation activates the SnRK2–ABI5/ABF signaling cascade [181]. The transcription factors *ABI5* (ABA-insensitive 5) and *ABFs* (ABA-responsive element binding factors) bind directly to ABA-responsive cis-elements (ABREs) in *PAL* promoters. For example, in banana, MaABI5-like directly interacts with the promoters of *MaPAL-like* and *MaPAL-like1*, activating their expression and channeling carbon flux toward flavonoid biosynthesis to enhance cold resistance [182]. Emerging evidence suggests that ABA-PAL crosstalk may include a positive feedback loop, in which PAL-derived chlorogenic acid further amplifies ABA signaling [183]. Consistent with this, increased ABA levels, elevated proline and phenolic contents, and enhanced PAL activity were observed in *Festulolium* leaves during pre-hardening and cold acclimation [184].

Under combined stresses, the interaction between ABA-PAL signaling and other phytohormonal pathways, particularly those mediated by SA and MeJA, forms a multilayered regulatory system. These hormones may act synergistically or antagonistically to fine-tune PAL activity, allowing plants to develop balanced and context-dependent responses to simultaneous stressors. The functional outcome varies with plant species, stress combinations, and relative hormone concentrations, and the underlying molecular mechanisms remain only partially understood. A defining feature of combined stresses—such as drought plus heat, drought plus pathogen attack, or flooding plus herbivory—is their ability to induce unique sets of stress-responsive genes that are not activated under individual stress conditions. These genes frequently include pathogenesis-related (PR) proteins and multiple PAL isoforms, both of which contribute to defense activation and metabolic reprogramming [185]. In maize (*Zea mays*), among nine putative PAL isoforms, RT-qPCR analyses have shown that *ZmPAL5*, *ZmPAL7*, *ZmPAL8*, and *ZmPAL9*, which cluster together phylogenetically, are specifically induced by combined stresses, with *ZmPAL7* and *ZmPAL8* showing the most pronounced responses [186,187]. In *A. thaliana*, sequential drought followed by pathogen infection strongly regulates the expression of hormone-biosynthetic genes, including ABA-related *NCED3* and *ABA3*, as well as *PAL4*. In contrast, combined drought and non-host pathogen stress upregulates SA biosynthesis genes (*EDS16*, *MES9*) alongside *PAL4* [188].

PAL plays a central role in these adaptive responses by mediating the biosynthesis of secondary metabolites that protect against oxidative damage. Many of these metabolites originate from phenylalanine and tyrosine and are synthesized via the PAL-mediated phenylpropanoid pathway [189]. Exogenous application of MeJA and SA enhances PAL activity in *Citrus sinensis*, improving tolerance to combined salt and pathogen stress [190]. Similarly, in plants subjected simultaneously to *Spodoptera frugiperda* herbivory and flooding, increased SA accumulation may result from the upregulation of *PAL* genes, although additional metabolic pathways, including upstream flavonoid derivatives, may also contribute [186,187].

Collectively, integration of ABA, SA, and MeJA signaling with PAL activity under combined stresses allows plants to prioritize carbon allocation toward functional metabolites, balancing defense against biotic challenges with adaptation to abiotic constraints. Although substantial progress has been made in defining this regulatory network, further research is needed to clarify the molecular mechanisms underlying hormone crosstalk, particularly concentration-dependent synergism and antagonism, and to determine the specific roles of individual PAL isoforms. Such insights will advance our understanding of multi-stress adaptation and support the development of crops with improved resilience to complex environmental fluctuations.

## 5. Summary and Perspectives

**Figure 5:**
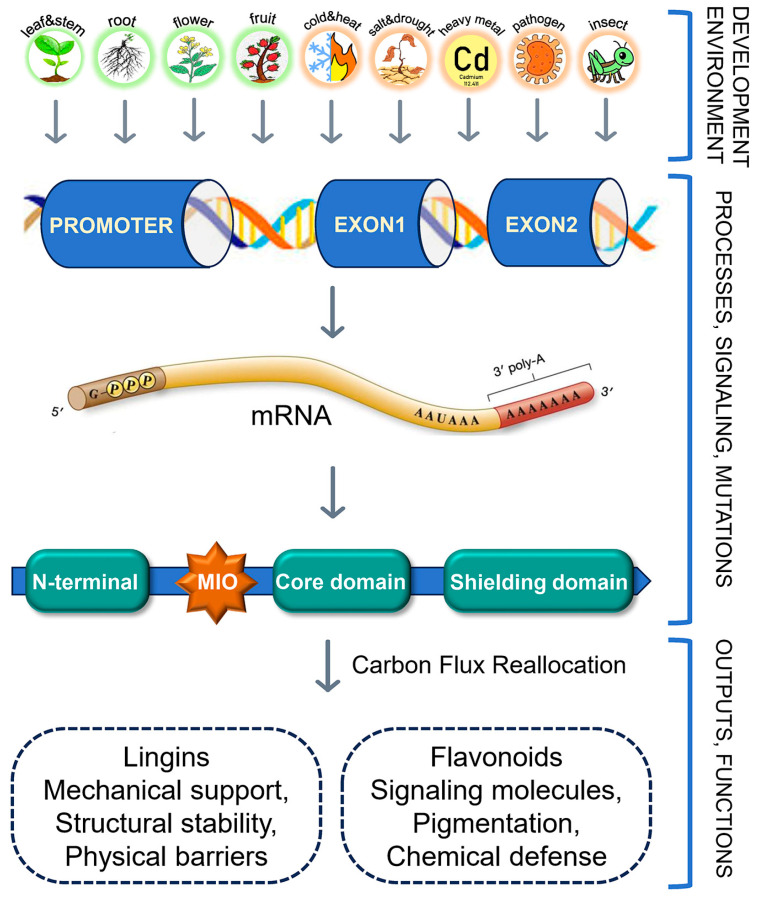
Schematic representation of the multilevel regulation of the *PAL* gene. Environmental and developmental cues, including organ development (stems, leaves, roots, flowers and fruits), abiotic stresses (temperature fluctuations, salinity, drought, heavy metal exposure), and biotic stresses (pathogen infection and herbivory), collectively modulate *PAL* gene expression. Upon activation, the PAL enzyme governs the redistribution of carbon flux from primary metabolism to the phenylpropanoid pathway, leading to the biosynthesis of key downstream metabolites. These include lignin, which reinforces mechanical strength and structural integrity, and flavonoids, which function as signaling molecules in pigmentation and chemical defense.

This review, based on evidence from algae, bryophytes, gymnosperms, and angiosperms, systematically elucidates the molecular basis, functional core, and regulatory principles of PAL-mediated carbon flux reallocation in plants. At the molecular level, PAL functions as a homotetramer, in which the MIO prosthetic group and key residues such as Ser and His collectively determine catalytic efficiency. Multiple regulatory layers—including post-translational modifications, epigenetic mechanisms, and phytohormone-PAL crosstalk (ABA, SA, MeJA)—form an integrated network to ensure precise dynamic allocation of carbon flux. The *PAL* gene family shows an evolutionary trajectory of “single-copy expansion–multi-copy specialization.” Lower plants harbor only 1–2 copies, primarily involved in basal phenylpropanoid metabolism, whereas angiosperms contain 3–15 copies with spatiotemporal expression patterns leading to functional diversification, thereby providing flexibility for carbon flux regulation. At the functional core, PAL acts as a “valve” between primary and secondary carbon metabolism, reallocating carbon flux to coordinate plant growth, development, and stress responses. During development, PAL-mediated lignin biosynthesis enhances root absorption efficiency and stem mechanical strength, while anthocyanin and flavonoid synthesis supports flower coloration and fruit quality. Under stress conditions, flavonoids scavenge ROS, lignin reinforces physical barriers, and phytoalexins suppress pathogens, together establishing a multidimensional defense system (Figure 5). Furthermore, PAL modulates plant growth by balancing carbon-nitrogen metabolism, and phytohormone-PAL crosstalk further tailors stress responses to specific environmental challenges. To provide a concise overview of PAL’s functional diversity across plant lineages, key findings from published studies are compiled in Supplementary Table S2.

Despite substantial progress, three major knowledge gaps remain. First, the fine-scale mechanisms of carbon flux control are poorly understood. Current studies largely focus on PAL activity or expression alone, lacking insights into its coordination with downstream enzymes such as C4H and 4CL, as well as quantitative analyses of dynamic carbon flux. Second, the mechanisms of phytohormone-PAL crosstalk under combined stresses are poorly understood. Under combined stresses such as “drought + pathogen infection + low nitrogen,” how PAL integrates signals such as SA and ABA to prioritize carbon flux allocation is not yet fully elucidated. Third, evolutionary molecular evidence is insufficient. The structural variation and functional divergence of PAL among plant lineages lack comprehensive cross-species analyses to uncover their evolutionary drivers. PAL functions as a central hub for carbon flux reallocation and stress signaling integration, and further exploration of its roles will provide a theoretical foundation for elucidating the mechanisms underlying the growth–defense trade-off in plants while also offering valuable insights for crop metabolic engineering. For instance, in agricultural applications, editing of *PAL* gene copies may optimize carbon allocation, supporting the breeding of crops with the combined traits of high yield, strong stress resistance, and superior quality.

## Data Availability

All data generated or analyzed during this study were included in this published article.

## Supplementary Materials

The following supporting information can be downloaded at https://www.mdpi.com/article/10.3390/plants14243811/s1, Table S1: Number of *Phenylalanine Ammonia-Lyase* (*PAL*) Genes in Different Plant Species and Related References [32,39,54,59,61,62,71,86,110,135,159,191,192,193,194,195,196,197,198,199,200,201,202,203,204,205,206]; Table S2: Functions, Species Distribution, and Related References of Plant *PAL* Genes [2,14,26,30,51,64,83,150,155,160,207,208,209,210,211,212,213,214,215,216,217,218,219,220,221,222,223,224,225,226,227,228,229,230,231,232,233].

## Abbreviations

The following abbreviations are used in this manuscript:

3-PGA	3-phosphoglycerate
4CL	4-coumarate-CoA ligase
ABA	abscisic acid
C4H	cinnamate 4-hydroxylase
CBSMs	carbon-based secondary metabolites
CCR	cinnamoyl-CoA reductase
*kcat*	turnover number
*Km*	Michaelis constant
MDA	malondialdehyde
MIO	4-methylidene-imidazole-5-one
PAL	phenylalanine ammonia-lyase
RNAi	RNA interference
ROS	reactive oxygen species
SA	salicylic acid
TCA	tricarboxylic acid
WGD	whole-genome duplication
