# Supplementary material for: Phenylalanine Ammonia-Lyase: A Core Regulator of Plant Carbon Metabolic Flux Redistribution—From Molecular Mechanisms and Growth Modulation to Stress Adaptability
Source: Plants (Basel). 2025 Dec 14;14(24):3811. doi: 10.3390/plants14243811 (PMC12737037; doi:10.3390/plants14243811)
Supplement: Supplementary file 1 [file plants-14-03811-s001.zip › plants-3979627-supplementary.pdf]

Supplementary Table 1 Number of Phenylalanine Ammonia-Lyase (PAL) Genes in Different Plant Species and Related References

Species	Number of PALs	Reference
Apostasia shenzhenica	5	Vishwakarma et al.,2023 [1]
Arachis hypogaea	10	Chai et al.,2024 [2]
Bambusa oldhamii	4	Hsieh et al.,2010 [3]
Brachypodium distachyon	8	Cass et al.,2015 [4]
Brassica oleracea	9	Karamat et al.,2024 [5]
Brassica rapa	7	Karamat et al.,2024 [5]
Brassica napus	17	Zhang et al.,2023 [6]
Citrullus lanatus	12	Dong and Shang, 2013 [7]
Citrus sinensis	41	Yin et al.,2024 [8]
Citrus reticulata	4	Yin et al.,2024 [8]
Citrus limon	4	Yin et al.,2024 [8]
Citrus grandis cv. 'Wanbaiyou'	4	Yin et al.,2024 [8]
Citrus grandis cv. Cupi Majiayou'	5	Yin et al.,2024 [8]
Citrus medica	5	Yin et al.,2024 [8]
Citrus clementina	5	Yin et al.,2024 [8]
Citrus ichangensis	5	Yin et al.,2024 [8]
Citrus australis	5	Yin et al.,2024 [8]
Cucumis sativus	11	Amjad et al.,2024 [9]
Cucumis sativus	15	Gu et al.,2024 [10]
Cynara cardunculus var. scolymus	3	de Paolis et al.,2008[11]
Dendrobium catenatum	2	Vishwakarma et al.,2023 [1]
Epimedium Pubescens	7	Xu et al.,2024 [12]
Ferula pseudalliacea	3	Shahidi et al.,2024 [13]
Hevea brasiliensis	8	Liu et al.,2024 [14]
Juglans regia	12	Yan et al.,2019 [15]
Juglans nigra	7	Zhou et al.,2025 [16]
Lithospermum erythrorhizon	2	Yazaki et al.,1997 [17]
Phalaenopsis equestris	2	Vishwakarma et al.,2023 [1]
Pinus banksiana	5	Butland et al.,1998 [18]
Populus kitakamiensis	4	Osakabe et al.,1995 [19]
Salix viminalis	5	de Jong et al.,2015 [20]
Solanum lycopersicum	14	Zhang et al.,2023 [21]
Solanum pennellii	11	Zhang et al.,2023 [21]
Solanum tuberosum	14	Mo et al.,2022 [22]
Sorghum bicolor	8	Jun et al.,2018 [23]
Triticum aestivum	37	Rasool et al.,2021 [24]
Triticum aestivum	54	Zhan et al.,2022 [25]
Vanilla planifolia	6	Kaur et al.,2025 [26]
Vaccinium dunalianum	7	An et al.,2025 [27]

Supplementary Table 2 Functions, Species Distribution, and Related References of Plant PAL Genes

Gene name	Species	Function	Reference
BcPAL1/2	*Brassica campestris*	Thermotolerance enhancement via activation by BcWRKY23/BcWRKY25	Gao et al.,2025 [28]
OsPAL1	*Oryza sativa*	Contribution to rice drought resistance and promotion of lignin and flavonoid accumulation	Xiang et al.,2025 [29]
GsPAL	*Gymnema sylvestre*	Key enzyme in flavonoid biosynthesis	Kalariya et al.,2024 [30]
ZzPAL	*Zingiber zerumbet*	Mediation of resistance against Pythium myriotylum in a SA-dependent manner and promotion of phenolic acid and lignin accumulation for cell wall reinforcement	Augustine et al.,2024 [31]
CsPAL	*Citrus sinensis*	Enhancement of citrus resistance to Penicillium digitatum via activation by CsWRKY33 and increased lignin and H_2O_2 accumulation	Wang et al.,2023 [32]
AmPAL	*Astragalus membranaceus*	Response to salt (NaCl) and saline-alkali ($NaHCO_3$) stresses, and increase in SOD activity, proline and chlorophyll contents in mature plants	Fan et al.,2022 [33]
FuPAL1	*Fritillaria unibracteata*	Enhancement of Arabidopsis drought tolerance via increased lignin and salicylic acid contents	Qin et al.,2022 [34]
AfPAL<br>ArPAL	*Anoectochilus formosanus*<br>*Anoectochilus roxburghii*	Response to L-phenylalanine, NaCl, UV and red light, and increase in flavonoid and anthocyanin contents	Yang et al.,2022 [35]
LcPAL1	*Lycium chinense*	Key gene in fruit flavonoid metabolism and promotion of flavonoid (anthocyanin, catechin) accumulation	Qiao et al.,2022 [36]
OsPAL	*Oryza sativa*	Reduction of rice Cd accumulation via stabilization by AetSRG1, promotion of SA synthesis, decrease in Cd^{2}+ flux, enhancement of SOD activity, and reduction of electrolyte leakage	Wei et al.,2022 [37]
BdPAL1	*Brachypodium distachyon*	Participation in antiviral defense via regulation of salicylic acid accumulation and defense gene expression	Pant et al.,2021 [38]
AtPAL1/2	*Arabidopsis thaliana*	Response to nitrogen deficiency and low temperature, and promotion of flavonoid (quercetin, anthocyanin) accumulation under low temperature	Olsen et al.,2008 [39]
LjPAL2	*Lonicera japonica*	Regulation of chlorogenic acid (CGA) biosynthesis	Zha et al.,2017 [40]
CaPAL1	*Capsicum annuum*	Positive regulation of SA-dependent defense signaling pathway and enhancement of pathogen (e.g., Xanthomonas campestris pv. vesicatoria) resistance	Kim and Hwang, 2014 [41]
GmPAL	*Glycine max*	Mediation of soybean heavy metal (Cd, Pb) detoxification and enhancement of lignin accumulation	Pawlak-Sprada et al.,2011 [42]
GmPAL	*Glycine max*	Main source of SA synthesis under pathogen induction, and enhancement of SA content and disease resistance	Shine et al.,2016 [43]
OsPAL	*Oryza sativa*	Response to Pyricularia oryzae and its elicitor	Wang et al.,2004 [44]
PkPAL1	*Picrorhiza kurrooa*	Response to biotic and abiotic elicitors	Bhat et al.,2014 [45]
CtPAL	*Carthamus tinctorius*	Response to SA, wounding and salt stress, and participation in flavonoid biosynthesis	Dehghan et al.,2014 [46]

Gene name	Species	Function	Reference
CsaPAL	*Capsicum annuum*	Key enzyme in capsaicin biosynthesis under drought stress	Phimchan et al.,2014 [47]
PePAL	*Phyllostachys edulis*	Involvement in bamboo lignin biosynthesis	Gao et al.,2012 [48]
JcPAL1	*Jatropha curcas*	Response to ABA, GA3, and high and low temperatures	Gao et al.,2012 [49]
SlPAL	*Solanum lycopersicum*	Enhancement of flavonoid accumulation and improvement of fruit quality	Maroga et al.,2019 [50]
AgPAL	*Angelica gigas*	Response to MeJA induction and association with coumarin biosynthesis	Park et al.,2010 [51]
SlPAL5	*Solanum lycopersicum*	Response to salt, mannitol, and low temperature	Guo and Wang, 2009 [52]
PtaPAL	*Pinus taeda*	Regulation of xylem-specific expression and bending stress response	Osakabe et al.,2009 [53]
RsPALrs1	*Rhodiola sachalinensis*	Modulation of phenylpropanoid metabolic flux and enhancement of p-coumaric acid accumulation	Ma et al.,2008 [54]
HvPAL	*Hordeum vulgare*	Participation in barley rust resistance and association with phenol and lignin biosynthesis	Prats et al.,2007 [55]
AiPAL	*Azolla imbricata*	Response to Cd stress and enhancement of Cd detoxification via promotion of anthocyanin accumulation	Dai et al.,2006 [56]
CicPAL	*Cicer arietinum*	Promotion of phenolic (tannic acid, gallic acid) accumulation and enhancement of resistance to Sclerotinia sclerotiorum	Basha et al.,2006 [57]
AsPAL	*Asparagus officinalis*	Response to harvest wounding and association with fiber development	Bhowmik and Matsui, 2005 [58]
DcPAL1	*Daucus carota*	Response to fungal elicitor, UV-B and dilution effect	Maeda et al.,2005[59]
CmPAL	*Citrus clementina*	Reduction of citrus chilling injury and improvement of cold resistance	Lafuente et al.,2004 [60]
GmPAL	*Glycine max*	Promotion of phenol accumulation	Khan et al.,2003 [61]
RaPAL	*Raphanus sativus*	Modulation of lignin biosynthesis and S/G ratio	Chen and McClure, 2000 [62]
TcPAL	*Taxus cuspidata*	Association with Taxol biosynthesis	Brincat et al.,2002 [63]
CmPAL	*Citrus clementina*	Association with cold resistance	Sanchez-Ballesta et al.,2000 [64]

Reference:

1. Vishwakarma, S.K.; Singh, N.; Kumaria, S. Genome-Wide Identification and Analysis of the PAL Genes from the Orchids Apostasia Shenzhenica, Dendrobium Catenatum and Phalaenopsis Equestris. *J Biomol Struct Dyn* **2023**, *41*, 1295–1308, doi:10.1080/07391102.2021.2019120.
2. Chai, P.; Cui, M.; Zhao, Q.; Chen, L.; Guo, T.; Guo, J.; Wu, C.; Du, P.; Liu, H.; Xu, J.; et al. Genome-Wide Characterization of the Phenylalanine Ammonia-Lyase Gene Family and Their Potential Roles in Response to Aspergillus Flavus L. Infection in Cultivated Peanut (Arachis Hypogaea L.). *Genes* **2024**, *15*, 265, doi:10.3390/genes15030265.
3. Hsieh, L.S.; Ma, G.J.; Yang, C.C.; Lee, P.D. Cloning, Expression, Site-Directed Mutagenesis and Immunolocalization of Phenylalanine Ammonia-Lyase in Bambusa Oldhamii. *Phytochemistry* **2010**, *71*, 1999–2009, doi:10.1016/j.phytochem.2010.09.019.
4. Cass, C.L.; Peraldi, A.; Dowd, P.F.; Mottiar, Y.; Santoro, N.; Karlen, S.D.; Bukhman, Y.V.; Foster, C.E.; Thrower, N.; Bruno, L.C.; et al. Effects of PHENYLALANINE AMMONIA LYASE (PAL) Knockdown on Cell Wall Composition, Biomass Digestibility, and Biotic and Abiotic Stress Responses in Brachypodium. *J Exp Bot* **2015**, *66*, 4317–4335, doi:10.1093/jxb/erv269.
5. Karamat, U.; Guo, J.; Jiang, S.; Khan, I.; Lu, M.; Fu, M.; Li, G. Comprehensive, Genome-Wide Identification and Expression Analyses of Phenylalanine Ammonia-Lyase Family under Abiotic Stresses in Brassica Oleracea. *Int. J. Mol.*

Sci. **2024**, *25*, 10276, doi:10.3390/ijms251910276.
6. Zhang, H.; Zhang, X.; Zhao, H.; Hu, J.; Wang, Z.; Yang, G.; Zhou, X.; Wan, H. Genome-Wide Identification and Expression Analysis of Phenylalanine Ammonia-Lyase (PAL) Family in Rapeseed (Brassica Napus L.). *BMC Plant Biol.* **2023**, *23*, 481–481, doi:10.1186/s12870-023-04472-9.
7. Dong, C.J.; Shang, Q.M. Genome-Wide Characterization of Phenylalanine Ammonia-Lyase Gene Family in Watermelon (Citrullus Lanatus). *Planta* **2013**, *238*, 35–49, doi:10.1007/s00425-013-1869-1.
8. Yin, T.; Xu, R.; Zhu, L.; Yang, X.; Zhang, M.; Li, X.; Zi, Y.; Wen, K.; Zhao, K.; Cai, H.; et al. Comparative Analysis of the PAL Gene Family in Nine Citruses Provides New Insights into the Stress Resistance Mechanism of Citrus Species. *BMC Genomics* **2024**, *25*, 1020, doi:10.1186/s12864-024-10938-3.
9. Amjad, M.; Wang, Y.; Han, S.; Haider, M.Z.; Sami, A.; Batool, A.; Shafiq, M.; Ali, Q.; Dong, J.; Sabir, I.A.; et al. Genome Wide Identification of Phenylalanine Ammonia-Lyase (PAL) Gene Family in Cucumis Sativus (Cucumber) against Abiotic Stress. *BMC Genom Data* **2024**, *25*, 76, doi:10.1186/s12863-024-01259-1.
10. Gu, J.; Sohail, H.; Qiu, L.; Chen, C.; Yue, H.; Li, Z.; Yang, X.; Zhang, L. Genome-Wide Characterization and Expression Analysis of CsPALs in Cucumber (Cucumis Sativus L.) Reveal Their Potential Roles in Abiotic Stress and Aphid Stress Tolerance. *Plants Basel* **2024**, *13*, doi:10.3390/plants13182537.
11. De Paolis, A.; Pignone, D.; Morgese, A.; Sonnante, G. Characterization and Differential Expression Analysis of Artichoke Phenylalanine Ammonia-Lyase-Coding Sequences. *Physiol Plant* **2008**, *132*, 33–43, doi:10.1111/j.1399-3054.2007.00996.x.
12. Xu, C.; Fan, X.; Shen, G.; Guo, B. Genome-Wide Identification of the Phenylalanine Ammonia-Lyase Gene from Epimedium Pubescens Maxim. (Berberidaceae): Novel Insight into the Evolution of the PAL Gene Family. *BMC Plant Biol.* **2024**, *24*, 831, doi:10.1186/s12870-024-05480-z.
13. Shahidi, P.; Bahramnejad, B.; Vafaee, Y.; Dastan, D.; Heidari, P. Isolation and Characterization of Phenylalanine Ammonia Lyase (PAL) Genes in Ferula Pseudalliacea: Insights into the Phenylpropanoid Pathway. *Genes* **2024**, *15*, 771, doi:10.3390/genes15060771.
14. Liu, H.; He, Q.; Hu, Y.; Lu, R.; Wu, S.; Feng, C.; Yuan, K.; Wang, Z. Genome-Wide Identification and Expression Profile Analysis of the Phenylalanine Ammonia-Lyase Gene Family in Hevea Brasiliensis. *Int J Mol Sci* **2024**, *25*, doi:10.3390/ijms25095052.
15. Yan, F.; Li, H.; Zhao, P. Genome-Wide Identification and Transcriptional Expression of the PAL Gene Family in Common Walnut (Juglans Regia L.). *Genes* **2019**, *10*, 46, doi:10.3390/genes10010046.
16. Zhou, H.; Liu, H.; Ma, J.; Yue, M.; Wang, Y.; Zhao, P.; Chen, Z. Genome-Wide Identification, Transcriptome Dynamics, and Expression Regulation of the Key Lignin Biosynthesis Gene Families PAL and CAD in Black Walnut Shell. *BMC Plant Biol* **2025**, *25*, 859, doi:10.1186/s12870-025-06884-1.
17. Yazaki, K.; Kataoka, M.; Honda, G.; Severin, K.; Heide, L. cDNA Cloning and Gene Expression of Phenylalanine Ammonia-Lyase in Lithospermum Erythrorhizon. *Biosci Biotechnol Biochem* **1997**, *61*, 1995–2003, doi:10.1271/bbb.61.1995.
18. Butland, S.L.; Chow, M.L.; Ellis, B.E. A Diverse Family of Phenylalanine Ammonia-Lyase Genes Expressed in Pine Trees and Cell Cultures. *Plant Mol Biol* **1998**, *37*, 15–24, doi:10.1023/a:1005941228246.
19. Osakabe, Y.; Osakabe, K.; Kawai, S.; Katayama, Y.; Morohoshi, N. Characterization of the Structure and Determination of mRNA Levels of the Phenylalanine Ammonia-Lyase Gene Family from Populus Kitakamiensis. *Plant Mol Biol* **1995**, *28*, 1133–1141, doi:10.1007/BF00032674.
20. de Jong, F.; Hanley, S.J.; Beale, M.H.; Karp, A. Characterisation of the Willow Phenylalanine Ammonia-Lyase (PAL) Gene Family Reveals Expression Differences Compared with Poplar. *Phytochemistry* **2015**, *117*, 90–97, doi:10.1016/j.phytochem.2015.06.005.
21. Zhang, F.; Wang, J.; Li, X.; Zhang, J.; Liu, Y.; Chen, Y.; Yu, Q.; Li, N. Genome-Wide Identification and Expression Analyses of Phenylalanine Ammonia-Lyase Gene Family Members from Tomato (Solanum Lycopersicum) Reveal Their Role in Root-Knot Nematode Infection. *Front Plant Sci* **2023**, *14*, 1204990, doi:10.3389/fpls.2023.1204990.
22. Mo, F.; Li, L.; Zhang, C.; Yang, C.; Chen, G.; Niu, Y.; Si, J.; Liu, T.; Sun, X.; Wang, S.; et al. Genome-Wide Analysis and Expression Profiling of the Phenylalanine Ammonia-Lyase Gene Family in Solanum Tuberosum. *Int J Mol Sci* **2022**, *23*, doi:10.3390/ijms23126833.

23. Jun, S.Y.; Sattler, S.A.; Cortez, G.S.; Vermerris, W.; Sattler, S.E.; Kang, C. Biochemical and Structural Analysis of Substrate Specificity of a Phenylalanine Ammonia-Lyase. *Plant Physiol* **2018**, *176*, 1452–1468, doi:10.1104/pp.17.01608.
24. Rasool, F.; Uzair, M.; Naeem, M.K.; Rehman, N.; Afroz, A.; Shah, H.; Khan, M.R. Phenylalanine Ammonia-Lyase (PAL) Genes Family in Wheat (Triticum Aestivum L.): Genome-Wide Characterization and Expression Profiling. *Agronomy* **2021**, *11*, 2511, doi:10.3390/agronomy11122511.
25. Zhan, C.; Li, Y.; Li, H.; Wang, M.; Gong, S.; Ma, D.; Li, Y. Phylogenomic Analysis of Phenylalanine Ammonia-Lyase (PAL) Multigene Family and Their Differential Expression Analysis in Wheat (Triticum Aestivum L.) Suggested Their Roles during Different Stress Responses. *Front Plant Sci* **2022**, *13*, 982457, doi:10.3389/fpls.2022.982457.
26. Kaur, A.; Sharma, K.; Pawar, S.V.; Sembi, J.K. Genome-Wide Characterization of PAL, C4H, and 4CL Genes Regulating the Phenylpropanoid Pathway in Vanilla Planifolia. *Sci Rep* **2025**, *15*, 10714, doi:10.1038/s41598-024-81968-w.
27. An, X.; Li, G.; Chen, A.; Zhao, P.; Ding, Y. Identification and Expression Analysis of PAL Genes Related to Chlorogenic Acid Synthesis in Vaccinium Dunalianum Wight. *Front. Plant Sci.* **2025**, *16*, 1544303, doi:10.3389/fpls.2025.1544303.
28. Gao, Z.; Wang, H.; Chen, X.; Ding, Q.; Li, E.; Shen, Y.; Jiang, C.; Li, Y.; Zhang, C.; Hou, X. BcVQ11A-BcWRKY23-BcWRKY25 Module Is Involved in Thermotolerance by Regulating Phenylalanine Ammonia-Lyase Activity in Non-Heading Chinese Cabbage. *Plant Cell Environ.* **2025**, *48*, 2357–2376, doi:10.1111/pce.15301.
29. Xiang, D.; Tu, H.; Yuan, Y.; Yao, Y.; Liao, W.; Wang, H.; Yan, Y.; Wang, Y.; Chen, Y.; Liu, D.; et al. A Blast-Resistant NLR Gene Confers Drought Resistance by Competitively Interacting with an E3 Ligase to Protect Phenylalanine Ammonia-Lyase in Rice. *Adv. Sci.* **2025**, *12*, e02662, doi:10.1002/advs.202502662.
30. Kalariya, K.A.; Mevada, R.R.; Das, M. Characterization of Phenylalanine Ammonia Lyase and Revealing Flavonoid Biosynthesis in Gymnema Sylvestre R. Br through Transcriptomic Approach. *J. Genet. Eng. Biotechnol.* **2024**, *22*, 100344, doi:10.1016/j.jgeb.2023.100344.
31. Augustine, L.; Varghese, L.; Kappachery, S.; Ramaswami, V.M.; Surendrababu, S.P.; Sakuntala, M.; Thomas, G. Comparative Analyses Reveal a Phenylalanine Ammonia Lyase Dependent and Salicylic Acid Mediated Host Resistance in Zingiber Zerumbet against the Necrotrophic Soft Rot Pathogen Pythium Myriotylum. *Plant Sci.* **2024**, *340*, 111972, doi:10.1016/j.plantsci.2023.111972.
32. Wang, W.; Li, T.; Chen, J.; Zhang, X.; Wei, L.; Yao, S.; Zeng, K. A Self-Regulated Transcription Factor CsWRKY33 Enhances Resistance of Citrus Fruit to Penicillium Digitatum. *Postharvest Biol. Technol.* **2023**, *198*, 112267, doi:https://doi.org/10.1016/j.postharvbio.2023.112267.
33. Fan, L.; Shi, G.; Yang, J.; Liu, G.; Niu, Z.; Ye, W.; Wu, S.; Wang, L.; Guan, Q. A Protective Role of Phenylalanine Ammonia-Lyase from Astragalus Membranaceus against Saline-Alkali Stress. *Int J Mol Sci* **2022**, *23*, doi:10.3390/ijms232415686.
34. Qin, Y.; Li, Q.; An, Q.; Li, D.; Huang, S.; Zhao, Y.; Chen, W.; Zhou, J.; Liao, H. A Phenylalanine Ammonia Lyase from Fritillaria Unibracteata Promotes Drought Tolerance by Regulating Lignin Biosynthesis and SA Signaling Pathway. *Int J Biol Macromol* **2022**, *213*, 574–588, doi:10.1016/j.ijbiomac.2022.05.161.
35. Yang, L.; Li, W.C.; Fu, F.L.; Qu, J.; Sun, F.; Yu, H.; Zhang, J. Characterization of Phenylalanine Ammonia-Lyase Genes Facilitating Flavonoid Biosynthesis from Two Species of Medicinal Plant Anoectochilus. *PeerJ* **2022**, *10*, e13614, doi:10.7717/peerj.13614.
36. Qiao, F.; Zhang, K.; Zhou, L.; Qiu, Q.-S.; Chen, Z.; Lu, Y.; Wang, L.; Geng, G.; Xie, H. Analysis of Flavonoid Metabolism during Fruit Development of Lycium Chinense. *J. Plant Physiol.* **2022**, *279*, 153856, doi:10.1016/j.jplph.2022.153856.
37. Wei, J.; Liao, S.; Li, M.; Zhu, B.; Wang, H.; Gu, L.; Yin, H.; X, D. AetSRG1 Contributes to the Inhibition of Wheat Cd Accumulation by Stabilizing Phenylalanine Ammonia Lyase. *J Hazard Mater* **2022**, *428*, 128226, doi:10.1016/j.jhazmat.2022.128226.
38. Pant, S.R.; Irigoyen, S.; Liu, J.; Bedre, R.; Christensen, S.A.; Schmelz, E.A.; Sedbrook, J.C.; Scholthof, K.; Mandadi, K.K. Brachypodium Phenylalanine Ammonia Lyase (PAL) Promotes Antiviral Defenses against Panicum Mosaic Virus and Its Satellites. *mBio* **2021**, *12*, doi:10.1128/mBio.03518-20.
39. Olsen, K.M.; Lea, U.S.; Slimestad, R.; Verheul, M.; Lillo, C. Differential Expression of Four *Arabidopsis PAL* Genes; *PAL1* and *PAL2* Have Functional Specialization in Abiotic Environmental-Triggered Flavonoid Synthesis. *J. Plant Physiol.* **2008**, *165*, 1491–1499, doi:10.1016/j.jplph.2007.11.005.
40. Zha, L.; Liu, S.; Liu, J.; Jiang, C.; Yu, S.; Yuan, Y.; Yang, J.; Wang, Y.; Huang, L. DNA Methylation Influences Chlorogenic

Acid Biosynthesis in Lonicera Japonica by Mediating LjbZIP8 to Regulate Phenylalanine Ammonia-Lyase 2 Expression. *Front Plant Sci* **2017**, *8*, 1178, doi:10.3389/fpls.2017.01178.
41. Kim, D.S.; Hwang, B.K. An Important Role of the Pepper Phenylalanine Ammonia-Lyase Gene (PAL1) in Salicylic Acid-Dependent Signalling of the Defence Response to Microbial Pathogens. *J Exp Bot* **2014**, *65*, 2295–2306, doi:10.1093/jxb/eru109.
42. Pawlak-Sprada, S.; Arasimowicz-Jelonek, M.; Podgorska, M.; Deckert, J. Activation of Phenylpropanoid Pathway in Legume Plants Exposed to Heavy Metals. Part I. Effects of Cadmium and Lead on Phenylalanine Ammonia-Lyase Gene Expression, Enzyme Activity and Lignin Content. *Acta Biochim Pol* **2011**, *58*, 211–216.
43. Shine, M.B.; Yang, J.W.; El-Habbak, M.; Nagyabhyru, P.; Fu, D.Q.; Navarre, D.; Ghabrial, S.; Kachroo, P.; Kachroo, A. Cooperative Functioning between Phenylalanine Ammonia Lyase and Isochorismate Synthase Activities Contributes to Salicylic Acid Biosynthesis in Soybean. *New Phytol* **2016**, *212*, 627–636, doi:10.1111/nph.14078.
44. Wang, L.; An, C.; Qian, W.; Liu, T.; Li, J.; Chen, Z. Detection of the Putative Cis-Region Involved in the Induction by a Pyricularia Oryzae Elicitor of the Promoter of a Gene Encoding Phenylalanine Ammonia-Lyase in Rice. *Plant Cell Rep* **2004**, *22*, 513–518, doi:10.1007/s00299-003-0717-3.
45. Bhat, W.W.; Razdan, S.; Rana, S.; Dhar, N.; Wani, T.A.; Qazi, P.; Vishwakarma, R.; Lattoo, S.K. A Phenylalanine Ammonia-Lyase Ortholog (PkPAL1) from Picrorhiza Kurrooa Royle Ex. Benth: Molecular Cloning, Promoter Analysis and Response to Biotic and Abiotic Elicitors. *Gene* **2014**, *547*, 245–256, doi:10.1016/j.gene.2014.06.046.
46. Dehghan, S.; Sadeghi, M.; Poppel, A.; Fischer, R.; Lakes-Harlan, R.; Kavousi, H.R.; Vilcinskas, A.; Rahnamaeian, M. Differential Inductions of Phenylalanine Ammonia-Lyase and Chalcone Synthase during Wounding, Salicylic Acid Treatment, and Salinity Stress in Safflower, Carthamus Tinctorius. *Biosci Rep* **2014**, *34*, doi:10.1042/BSR20140026.
47. Phimchan, P.; Chanthai, S.; Bosland, P.W.; Techawongstien, S. Enzymatic Changes in Phenylalanine Ammonia-Lyase, Cinnamic-4-Hydroxylase, Capsaicin Synthase, and Peroxidase Activities in Capsicum under Drought Stress. *J Agric Food Chem* **2014**, *62*, 7057–7062, doi:10.1021/jf4051717.
48. Gao, Z.M.; Wang, X.C.; Peng, Z.H.; Zheng, B.; Liu, Q. Characterization and Primary Functional Analysis of Phenylalanine Ammonia-Lyase Gene from Phyllostachys Edulis. *Plant Cell Rep* **2012**, *31*, 1345–1356, doi:10.1007/s00299-012-1253-9.
49. Gao, J.; Zhang, S.; Cai, F.; Zheng, X.; Lin, N.; Qin, X.; Ou, Y.; Gu, X.; Zhu, X.; Xu, Y.; et al. Characterization, and Expression Profile of a Phenylalanine Ammonia Lyase Gene from Jatropha Curcas L. *Mol Biol Rep* **2012**, *39*, 3443–3452, doi:10.1007/s11033-011-1116-4.
50. Maroga, G.M.; Soundy, P.; Sivakumar, D. Different Postharvest Responses of Fresh-Cut Sweet Peppers Related to Quality and Antioxidant and Phenylalanine Ammonia Lyase Activities during Exposure to Light-Emitting Diode Treatments. *Foods* **2019**, *8*, doi:10.3390/foods8090359.
51. Park, J.H.; Park, N.I.; Xu, H.; Park, S.U. Cloning and Characterization of Phenylalanine Ammonia-Lyase and Cinnamate 4-Hydroxylase and Pyranocoumarin Biosynthesis in Angelica Gigas. *J Nat Prod* **2010**, *73*, 1394–1397, doi:10.1021/np1003356.
52. Guo, J.; Wang, M.H. Characterization of the Phenylalanine Ammonia-Lyase Gene (SlPAL5) from Tomato (Solanum Lycopersicum L.). *Mol Biol Rep* **2009**, *36*, 1579–1585, doi:10.1007/s11033-008-9354-9.
53. Osakabe, Y.; Osakabe, K.; Chiang, V.L. Characterization of the Tissue-Specific Expression of Phenylalanine Ammonia-Lyase Gene Promoter from Loblolly Pine (Pinus Taeda) in Nicotiana Tabacum. *Plant Cell Rep* **2009**, *28*, 1309–1317, doi:10.1007/s00299-009-0707-1.
54. Ma, L.Q.; Gao, D.Y.; Wang, Y.N.; Wang, H.H.; Zhang, J.X.; Pang, X.B.; Hu, T.S.; Lu, S.Y.; Li, G.F.; Ye, H.C.; et al. Effects of Overexpression of Endogenous Phenylalanine Ammonia-Lyase (PALrs1) on Accumulation of Salidroside in Rhodiola Sachalinensis. *Plant Biol Stuttg* **2008**, *10*, 323–333, doi:10.1111/j.1438-8677.2007.00024.x.
55. Prats, E.; Martinez, F.; Rojas-Molina, M.M.; Rubiales, D. Differential Effects of Phenylalanine Ammonia Lyase, Cinnamyl Alcohol Dehydrogenase, and Energetic Metabolism Inhibition on Resistance of Appropriate Host and Nonhost Cereal-Rust Interactions. *Phytopathology* **2007**, *97*, 1578–1583, doi:10.1094/PHYTO-97-12-1578.
56. Dai, L.P.; Xiong, Z.T.; Huang, Y.; Li, M.J. Cadmium-Induced Changes in Pigments, Total Phenolics, and Phenylalanine Ammonia-Lyase Activity in Fronds of Azolla Imbricata. *Env. Toxicol* **2006**, *21*, 505–512, doi:10.1002/tox.20212.
57. Basha, S.A.; Sarma, B.K.; Singh, D.P.; Annapurna, K.; Singh, U.P. Differential Methods of Inoculation of Plant

Growth-Promoting Rhizobacteria Induce Synthesis of Phenylalanine Ammonia-Lyase and Phenolic Compounds Differentially in Chickpea. *Folia Microbiol Praha* **2006**, *51*, 463–468, doi:10.1007/BF02931592.
58. Bhowmik, P.K.; Matsui, T. Changes in Phenylalanine Ammonia-Lyase Activity and Gene Expression during Storage of Asparagus Spears. *Z Naturforsch C J Biosci* **2005**, *60*, 128–132, doi:10.1515/znc-2005-1-223.
59. Maeda, K.; Kimura, S.; Demura, T.; Takeda, J.; Ozeki, Y. DcMYB1 Acts as a Transcriptional Activator of the Carrot Phenylalanine Ammonia-Lyase Gene (DcPAL1) in Response to Elicitor Treatment, UV-B Irradiation and the Dilution Effect. *Plant Mol Biol* **2005**, *59*, 739–752, doi:10.1007/s11103-005-0910-6.
60. Lafuente, M.T.; Sala, J.M.; Zacarias, L. Active Oxygen Detoxifying Enzymes and Phenylalanine Ammonia-Lyase in the Ethylene-Induced Chilling Tolerance in Citrus Fruit. *J Agric Food Chem* **2004**, *52*, 3606–3611, doi:10.1021/jf035185i.
61. Khan, W.; Prithiviraj, B.; Smith, D.L. Chitosan and Chitin Oligomers Increase Phenylalanine Ammonia-Lyase and Tyrosine Ammonia-Lyase Activities in Soybean Leaves. *J Plant Physiol* **2003**, *160*, 859–863, doi:10.1078/0176-1617-00905.
62. Chen, M.; McClure, J.W. Altered Lignin Composition in Phenylalanine Ammonia-Lyase-Inhibited Radish Seedlings: Implications for Seed-Derived Sinapoyl Esters as Lignin Precursors. *Phytochemistry* **2000**, *53*, 365–370, doi:10.1016/s0031-9422(99)00531-2.
63. Brincat, M.C.; Gibson, D.M.; Shuler, M.L. Alterations in Taxol Production in Plant Cell Culture via Manipulation of the Phenylalanine Ammonia Lyase Pathway. *Biotechnol Prog* **2002**, *18*, 1149–1156, doi:10.1021/bp0256115.
64. Sanchez-Ballesta, M.T.; Zacarias, L.; Granell, A.; Lafuente, M.T. Accumulation of PAL Transcript and PAL Activity as Affected by Heat-Conditioning and Low-Temperature Storage and Its Relation to Chilling Sensitivity in Mandarin Fruits. *J. Agric. Food Chem.* **2000**, *48*, 2726–2731, doi:10.1021/jf991141r.
